# A comprehensive review of human trophoblast fusion models: recent developments and challenges

**DOI:** 10.1038/s41420-023-01670-0

**Published:** 2023-10-10

**Authors:** Xia Li, Zhuo-Hang Li, Ying-Xiong Wang, Tai-Hang Liu

**Affiliations:** 1 Department of Bioinformatics, School of Basic Medical Sciences, Chongqing Medical University, 400016 Chongqing, China; 2 The Joint International Research Laboratory of Reproduction and Development, Ministry of Education, 400016 Chongqing, China; 3 Medical Laboratory Department, Traditional Chinese Medicine Hospital of Yaan, 625099 Sichuan, China

**Keywords:** Cell biology, Experimental models of disease

## Abstract

As an essential component of the maternal-fetal interface, the placental syncytiotrophoblast layer contributes to a successful pregnancy by secreting hormones necessary for pregnancy, transporting nutrients, mediating gas exchange, balancing immune tolerance, and resisting pathogen infection. Notably, the deficiency in mononuclear trophoblast cells fusing into multinucleated syncytiotrophoblast has been linked to adverse pregnancy outcomes, such as preeclampsia, fetal growth restriction, preterm birth, and stillbirth. Despite the availability of many models for the study of trophoblast fusion, there exists a notable disparity from the ideal model, limiting the deeper exploration into the placental development. Here, we reviewed the existing models employed for the investigation of human trophoblast fusion from several aspects, including the development history, latest progress, advantages, disadvantages, scope of application, and challenges. The literature searched covers the monolayer cell lines, primary human trophoblast, placental explants, human trophoblast stem cells, human pluripotent stem cells, three-dimensional cell spheres, organoids, and placenta-on-a-chip from 1938 to 2023. These diverse models have significantly enhanced our comprehension of placental development regulation and the underlying mechanisms of placental-related disorders. Through this review, our objective is to provide readers with a thorough understanding of the existing trophoblast fusion models, making it easier to select most suitable models to address specific experimental requirements or scientific inquiries.

**Figure:**
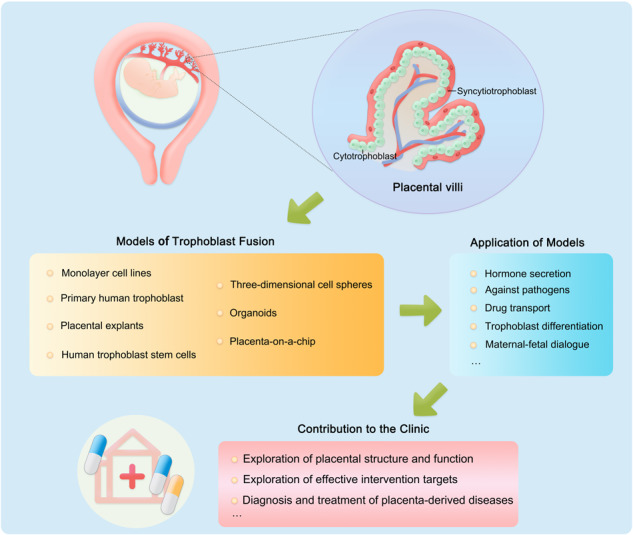
Establishment and application of the existing human placental trophoblast fusion models.

Establishment and application of the existing human placental trophoblast fusion models.

## Facts

- The process of cytotrophoblasts fusion into syncytiotrophoblast occurs throughout pregnancy.
- Impaired trophoblast cell fusion can lead to a variety of placenta-derived pregnancy diseases, such as preeclampsia, recurrent spontaneous abortion, and fetal growth restriction.
- Cytotrophoblasts encompass various cell subtypes, whereas reports on the subtypes of syncytiotrophoblast are relatively scarce.
- The similarity of among various trophoblast cell fusion models, including choriocarcinoma cell lines, primary trophoblast cells, trophoblast stem cells, organoids, and human trophoblasts, remains largely unexplored.

## Open questions

- How to confirm the heterogeneity between syncytiotrophoblast in different periods of pregnancy.
- How the maternal-fetal interface microenvironment interacts with trophoblasts to regulate the process of trophoblast fusion.
- What are the biggest challenges for the application of existing trophoblast fusion models in clinical practice.

## Introduction

The placenta, known as the tree of life, takes root in the fertile soil of the mother’s uterus, nourishing the growth of new life. The remarkable organ undergoes highly dynamic changes, coordinating with the mother to meet the evolving demands of fetal development during pregnancy. The continuous proliferation of cytotrophoblasts (CTBs) and their subsequent differentiation into multinucleated syncytiotrophoblast (STB) and extravillous trophoblast (EVT) is one of the critical factors for placental function. The STB is a terminal differentiated cell which can maintain its integrity only through the continuous fusion of CTBs. Additionally, the fusing CTBs replenishes the terminally differentiated STB with various components such as proteins, nucleic acids, lipids, and organelles to balance the apoptotic substances released by STB [1]. The STB forms a continuous surface layer of approximately 12–14 m^2^ area that covers the surface of placental villi and completely separates maternal blood from fetal circulation [2]. Moreover, the STB performs various biological functions, such as facilitating gas and nutrient exchange between the mother and fetus, regulating maternal-fetal immune tolerance, secreting various hormones to maintain pregnancy, and acting as the primary maternal-fetal barrier against pathogens. Studies have substantiated that aberrations in the formation of STB can lead to various placental dysfunction syndromes, such as preeclampsia (PE), fetal growth restriction (FGR), preterm birth, and stillbirth [3]. These adverse pregnancy outcomes bear lifelong impacts on the well-being of the mother and infant. Therefore, gaining an in-depth understanding of the mechanism of STB formation is crucial in addressing these issues.

One of the principal challenges in investigating the mechanism of trophoblast fusion lies in the absence of an ideal model. Human placental samples obtained post-delivery and placental villi from early pregnancy termination have emerged as favored models for placental research, owing to their ready accessibility. Nevertheless, acquiring placental samples during the second-trimester remains a formidable obstacle. Furthermore, the limited number and proliferation ability of isolated primary trophoblasts restricts their widespread use. Although animal models are commonly employed to study placental development and have been instrumental in advancing our collective understanding of placentation, there are key anatomical and physiological differences between species [4]. Thus, the exploration of the biological dynamics governing placental trophoblasts heavily relies on diverse in vitro models, encompassing both monolayer and three-dimensional (3D) cell models. Nonetheless, despite substantial progress, discernible gaps persist, impeding a comprehensive representation of the intricate structure and functionality of placental trophoblasts. In this study, we systematically evaluated the existing placental trophoblast fusion models, providing a comprehensive overview of their development history, applications, and limitations (Table 1). We hope that this will serve as a valuable reference for researchers in the selection of appropriate trophoblast fusion models.

**Table 1:** The overview of the existing models for investigating trophoblast fusion.

Models		Origin	Medium	Inducer	Advantages	Limitations	Reference(s)
Monolayer cell lines	BeWo	Choriocarcinoma	Ham’s F12, DMEM/F12	Forskolin, 8-bromo-cAMP	• Unlimited self-renewing capacity • Easy culture and stability • Easy genetic manipulation • Cell fusion and expression of fusion markers can be induced	• Tumor-derived and Chromosome aberration • Long-term in vitro culture is prone to mutation • Different genome-wide DNA methylation patterns with primary trophoblast	[21, 34,145]
	JEG-3		MEM, DMEM				[146]
	JAR		DMEM, RPMI 1640	Methotrexate			[147]
Primary human trophoblast cells (PHT)	NA	Term placenta, First-trimester placenta	DMEM/F12, DMEM	Forskolin, Spontaneous cell fusion	• Source of placenta • Physiological characteristics of spontaneous differentiation • Close to the actual situation in vivo • Cell fusion and expression of fusion markers can be induced	• Contamination with other cells • Limited proliferation • Hard genetic manipulation • Ethical issues	[5, 62, 70, 148,149]
Placental explants	NA		DMEM/F12, 199	Forskolin, Spontaneous cell fusion	• Source of placenta • Structure of retaining villi tissue • Physiological characteristics of spontaneous differentiation • Close to the actual situation in vivo • Cell fusion and expression of fusion markers can be induced	• Contamination with other cells • Limited proliferation • Hard genetic manipulation • Ethical issues	[80, 83,150]
Human trophoblast stem cells (TSCs)	TSCs		DMEM/F12	Forskolin, Spontaneous cell fusion	• Normal karyotypes • Similar gene expression profiles with PHT • Unlimited self-renewing capacity • Easy genetic manipulation • Cell fusion and expression of fusion markers can be induced	• Different DNA methylation patterns with primary trophoblast cells • Complex culturing system and high culturing cost	[20, 84,85]
	Human embryonic stem cells (ESCs)	blastocyst	mTeSR1	BMP4, Forskolin	• High differentiation potential • Unlimited self-renewing capacity • Easy genetic manipulation • Similar transcriptional and chromatin landscape characteristics to the blastocyst-derived TSC • Placenta-like CpG methylation patterns • Cell fusion and expression of fusion markers can be induced	• Heterogeneity of differentiation products • The system of culture and induction is not perfect • widespread imprint erasure • Different DNA methylation patterns with primary trophoblast cells	[99, 101, 103, 104, 151,152]
	Induced pluripotent stem cells (iPSCs)	Somatic cells	DMEM	Forskolin	• Source cells are easy to obtain • High differentiation potential • Cell fusion and expression of fusion markers can be induced	• widespread imprint erasure • Heterogeneity of differentiation products • The system of culture and induction is not perfect • High culturing cost	[109]
	Expanded potential stem cells (ePSCs)	ESCs, iPSCs, Somatic cells	DMEM/F-12	Forskolin	• High differentiation potential • Closer transcriptional pattern to early embryos compared with ESCs • Cell fusion and expression of fusion markers can be induced	• Heterogeneity of differentiation products • The system of culture and induction is not perfect • High culturing cost • hEPSC-derived cells exhibited higher expression of amniotic membrane-enriched genes	[108,153]
3D cell spheres	NA	JEG-3, TSCs, EBs	DMEM, GTSF-2	Forskolin, Spontaneous cell fusion	• 3D structure similar to placental villi • Transcriptional profiles similar to PHT • Easy genetic manipulation	• Long-term in vitro culture is prone to mutation • The unclear similarity between simulated 3D growth environment and in-vivo environment	[84, 112, 117,119]
Organoids	NA	placenta, PSCs, JEG-3	DMEM/F12	Spontaneous cell fusion	• Source of placenta • Physiological characteristics of spontaneous differentiation • The similar structure of villi tissue • similar features between the CTB-ORGs and the placental villi of the first-trimester	• STB is formed in the lumen of trophoblast-like ORGs • High culturing cost • only represent the expression profile of first-trimester placental tissue	[120–122,154]
Placenta-on-a-chip	NA	BeWo, PHT	NA	Forskolin	• Reduced consumption of cells, • Real-time detect cells and obtaining dynamic data • Multicellular co-culture • Precise control of culture microenvironment	• Need professional guidance, • High culturing cost	[130, 131,133]

^a^ NA, Not Available.

## Formation of syncytiotrophoblast subtypes

**Fig. 1:**
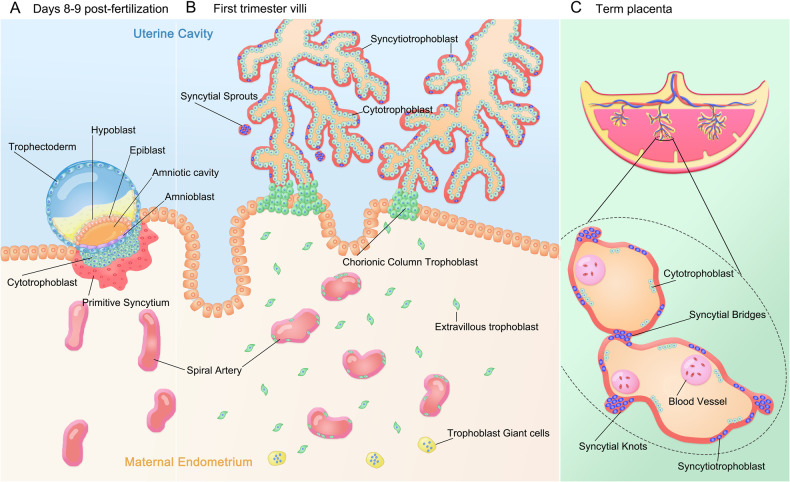
Formation of syncytiotrophoblast subtypes during dynamic development of the human placenta. **A** The blastocyst is implanted into the receptive maternal endometrium around days 5–7 post-fertilization. At days 8–9 post-fertilization, the trophectoderm differentiates into cytotrophoblasts (CTBs), which fuse to form a multinucleated primitive syncytium. The primitive syncytium has a strong invasive ability and can regulate maternal-fetal immune tolerance, serving as the front end of the invading maternal endometrium. As the embryo is fully implanted into the endometrium, the primitive syncytium will surround the embryo. **B** By early in the first-trimester, the placental villi wrapped by inner proliferative mononuclear CTBs and outer continuous multinucleated syncytiotrophoblast (STB) gradually formed. With the development of the placental villi, syncytial sprouts representing the formation of new villi fall off from the surface. By late the first-trimester, multistage branches of villous trees are established, and extravillous trophoblast (EVT) differentiated from CTBs invade the endometrium and myometrium deeply. The EVT also replaces vascular endothelium to remodel maternal spiral arteries. Additionally, multinucleated trophoblast giant cells (TGCs) are present in the depths of the decidua and myometrium, and most believe that they arise from differentiated interstitial EVT. **C** As the placenta develops, the CTB layer cells become discontinuous in the early second-trimester, and proliferative CTBs gradually decrease in the second/early term placenta, with most areas only covered by continuous STB. Placental vessels will be closer to the outer wall of the villi to facilitate material exchange. Syncytial knots on the surface of the villi and syncytial bridges connecting the villi are also present. Syncytial knots increase with the development of the placenta and are more common in pathological pregnancies with complications such as PE, FGR, and stillbirth.

Throughout embryonic development, two distinct subtypes of STB have been identified, the primitive syncytium and definitive STB (Fig. 1) [5]. Upon implantation of the blastocyst in the uterus around days 5–7 post-fertilization, the trophoblast fuses to form the primitive syncytium, which destroys capillaries and endometrial glands and secretes large amounts of human chorionic gonadotropin (hCG) as the frontier of embryo invasion [6]. Subsequently, the CTBs below the primitive syncytium rapidly proliferate and differentiate, protruding to generate primary villi, with the STB layer enveloping the villous surface. Secondary and tertiary villi emerge sequentially around days 17–18 post-fertilization, initiating the early development of placental villous trees [7]. The CTB layer cells become discontinuous in the early second-trimester and gradually decrease later in the second/early term placenta [8]. It is worth noting that the definitive STB undergoes dynamic changes throughout pregnancy to meet the growing needs of the fetus. Trophoblast single-cell and RNA-sequencing (RNA-seq) results have revealed substantial variations in gene expression patterns across different gestational ages, providing additional evidence of the dynamic changes of the definitive STB [9].

In addition to the dynamic expression pattern changes, the nuclei of STB also exhibit a range of morphologies. These nuclei can aggregate to form syncytial nuclear aggregates (SNAs), characterized by at least ten trophoblast nuclei gathering into clusters, including sprouts, bridges, and knots [10, 11]. Syncytial sprouts are typically observed in the first-trimester placenta and signify the initial stage of villi development [12]. Syncytial bridges are highly nucleated regions that connect the two villi, while syncytial knots form closer to the term and protrude slightly from the surface of the villi. An increased number of syncytial knots has been reported in the placenta of pregnancies with combined PE and FGR, whether this phenomenon reflects an exaggerated aging or apoptotic death of STB is still debated [13].

## Key steps and regulation of trophoblast fusion

The process of CTBs fuse to STB is closely modulated by myriad intrinsic and microenvironmental factors [14]. Currently, this process can be summarized in three stages [15]. (i) Competence: CTBs must first exit the mitotic cell cycle and differentiate into fusion-competent cells [16]. During this stage, certain progenitor cell maintenance factors, including *TEAD4* [17], *YAP* [18], *TP63*, *GATA3* [19], and *MSX2*, as well as signal pathways such as Wnt and activin/TGF-β, were suppressed. Meanwhile, other factors and signal pathways that drive STB formation were activated, including key transcription factors such as *GCM1* [20], *TFAP2A*, *OVOL1* [21], *CREB*, epigenetic regulators *HDACs*, and cAMP/PKA and MAPK pathways. (ii) Commitment: This stage is characterized by intercellular adhesion and communication processes that lead to the activation, expression, exposure, or assembly of fusogenic machinery. Intercellular adhesion is triggered by the aggregation of adjacent cells by adhesion junctions (E-cadherin, E-cad) and tight junctions [22] (zonula occludens-1, ZO-1) [23, 24]. Simultaneously, the initiation of gap junction (connexin-43, Cx43) communication leads to synchronization and signal exchange between cells. (iii) Cell fusion: The fusion related-proteins syncytin-1 and syncytin-2 mediated the opening of the fusion pore, completing the membrane merging and cell content mixing [25, 26]. These pivotal regulatory factors also serve as molecular markers for characterizing the differentiation process of each model (Table 2). Detailed insights into the characteristics of signaling pathways and factors involved in the trophoblast fusion process have been extensively reviewed elsewhere [27, 28].

**Table 2:** The molecular markers used for identifying the trophoblast fusion stage.

Models	Expression trend#	Pluripotency markers	Trophectoderm markers	Syncytiotrophoblast markers	References
BeWo	+	OCT4, SOX2, NANOG	CKT7, CK17, CK18, YAP, TP63, ID1, KI67, MMP19, ASCL2	hCG, GCM1, Syncytin-1, Syncytin2, PLAP, CYP11A1, Progesterone, Estradiol, OVOL1, ADAM12, P57, FLRG, TREML2, FURIN	[21, 23, 44,155]
	-	NA	Vimentin	E-Cadherin, ZO-1	[23,156]
JEG-3	+	SOX2, NANOG	KRT18, KI67	hCG	[44, 155,157]
	-	NA	Vimentin	E-Cadherin	[156,158]
JAR	+	NA	E-Cadherin, ZO-1, CK7	hCG	[157,158]
	-	NA	Vimentin	NA	[158]
PHT	+	NA	TEAD4,	hCG, GCM1, Syncytin-1, Syncytin2, PLAP, SDC1, ENDOU,	[23, 34, 41, 65,159]
	-	NA	NA	E-Cadherin	[23, 41,145]
Placental explants	+	NA	CK7, KI67	hCG, hPL, FURIN	[83,160]
	-	NA	Vimentin	NA	[161]
TSCs	+	NA	TEAD4, TFAP2C, GATA3, CK7, EGFR, YAP, TP63, ELF5, ITGA6, KI67	hCG, SDC1	[84, 85,152]
	-	NA	Vimentin	E-Cadherin, HLA-G	[84,85]
ESCs	+	OCT4, TERT, CD75, SUSD2, NANOG, TFAP2C	TEAD4, TFAP2C, GATA2, GATA3, CK7, CK18, EGFR, ELF5, TP63, ITGA6, EPCAM, HAVCR1, VGLL1	hCG, GCM1, PSG4, Progesterone, Estradiol, PGF, SDC1, TEAD3	[101, 103, 151,152]
	-	TP63, TFAP2C, GATA3	NANOG, OCT4, CDX2	NA	[101, 104,151]
iPSCs	+	OCT4, CDX2, SOX2, NANOG, rBC2LCN	TEAD4, TFAP2C, GATA2, GATA3, CK7, YAP, TP63, VGLL1, NR2F2	hCG, SDC1, DSP	[109, 162,163]
	-	TP63, GATA3	OCT4, SOX2, NANOG	NA	[162]
ePSCs	+	SOX17, BLIMP1, OCT4	CDX2, GATA2, GATA3, ELF5, KRT8, MCT1, CYP19A1, TFAP2C	hCG, GCM1, Syncytin-1, Syncytin2, P57, PD-L1, EGFR	[108,153]
	-	PAX6, GATA4, SOX7, PGF, TFAP2C	NA	NA	[108]
3D cell spheres	+	OCT4, SOX2, NANOG	TEAD4, TFAP2A, YAP, CYT19, ERBB2	hCG, hPL, PP13, MFSD2, Syncytin-1, p-CREB	[112, 119, 122,157]
	-	NA	NA	E-Cadherin, ZO-1	[112,113]
Organoids	+	OCT4	TEAD4, CDX2, TFAP2A, TFAP2C, GATA3, CK7, EGFR, TP63, ELF5, ITGA6, KI67, EPCAM, CCNA, CK17, CK23, CCNB1,	hCG, GCM1, Syncytin-1, hPL, CD71, CD46, SDC1, ENDOU, INSL4, KISS1, GDF15, MUC16, S100P	[120, 121, 154,164]
	-	NA	Vimentin	E-Cadherin	[120,154]
Placenta-on-a-chip	+	NA	EPCAM	hCG, GLUT1	[128, 131,134]
	-	NA	NA	E-Cadherin	[128,136]
villous tissue	+	NA	TEAD4, CK7, EGFR, YAP, TP63, KI67, PCNA	hCG, hPL, ADAM12, ENDOU, FURIN	[23, 65,165]
	-	NA	Vimentin	E-Cadherin	[23,166]

^a^In the process of trophoblast fusion, the up-regulated molecular markers are represented by “+“, and the down-regulated molecular markers are represented by “−“.

^b^NA, Not Available.

## Advances in existing trophoblast fusion models

**Fig. 2:**
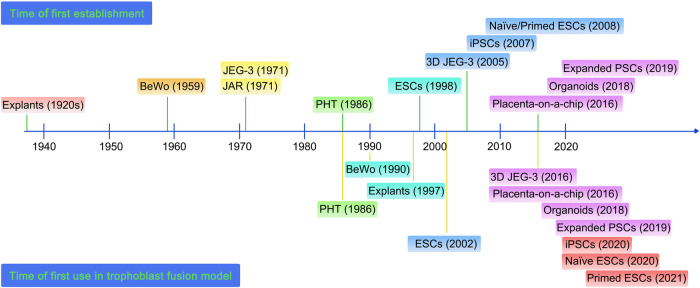
Timeline of the establishment of various cell lines and trophoblast fusion models. The timeline is divided into intervals of 10 years, with events in each decade marked with the same background box. The time of the first establishment of various cell lines is marked above the timeline, while the time of the first use of these cell lines in trophoblast fusion models is marked below the timeline.

**Fig. 3:**
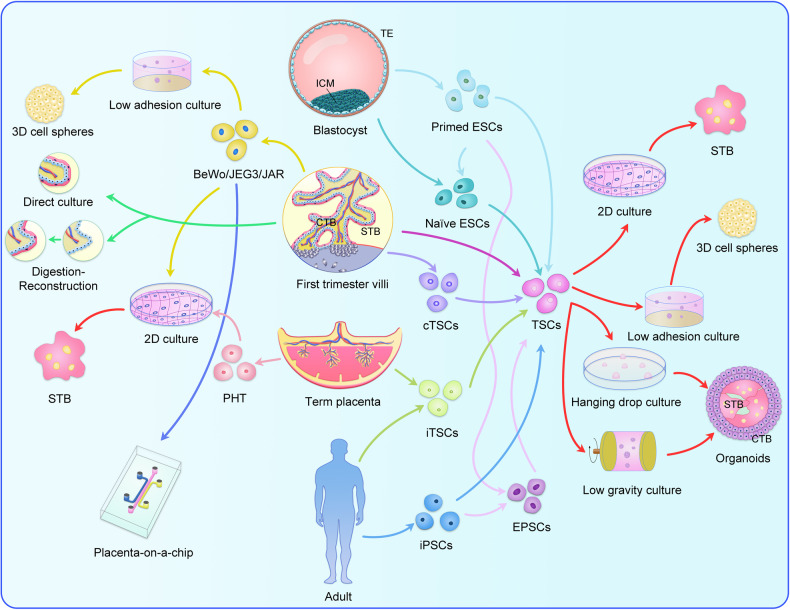
The overview of the schemes for the existing trophoblast fusion models. Choriocarcinoma-derived cell lines, BeWo, JEG-3, and JAR, can be induced to fuse into syncytiotrophoblast (STB) and three-dimensional (3D) cell spheres using Forskolin in monolayer culture and 3D low adhesion culture, respectively. These trophoblast cell lines can also be co-cultured with other cell types on microfluidic devices to create placenta-on-a-chip. Placental explants can generate new STB through direct culture or “digestion-reconstruction” culture in vitro. Primary human trophoblasts (PHT) isolated from term placenta can spontaneously fuse to form STB in monolayer culture. Human trophoblast stem cells (TSCs) can now be obtained through the induction of various stem cells or specific trophoblast culture methods, including primed embryonic stem cells (ESCs), Naïve ESCs, villi cytotrophoblast cells (vCTBs), human candidate TSCs population (cTSCs), induced TSCs (iTSCs), induced PSCs (iPSCs), and expanded PSCs (ePSCs). These TSCs can then be induced to differentiate into STB through monolayer culture or form organoids or 3D cell spheres using a range of 3D cultures, including low adhesion culture, hanging culture, and low gravity culture.

For an extended period, the placenta was referred to as a “forgotten organ”. It was not until the mid-20th century that researchers gradually shifted from descriptive studies of the placenta to mechanism research with a deep understanding of its crucial role in successful pregnancies. During this phase, various traditional placental cell lines were gradually generated and harnessed in placental research endeavors. However, it was not until the year 1986 that primary human trophoblast cells (PHTs) were first employed to establish the first trophoblast fusion model (Fig. 2). Over time, propelled by technological advancements and the accumulation of knowledge, researchers rapidly developed various trophoblast models, which were then used to generate trophoblast fusion models. In the last seven years, the trophoblast fusion models showed a blowout development, including placenta-on-a-chip, various human pluripotent stem cells (PSCs), and organoids (Fig. 3). These innovative models hold great promise for advancing placental research and breaking through current bottlenecks.

## Monolayer cell lines

Immortalized cell lines have long been the preferred choice for in vitro experiments due to their convenient acquisition, culture, and high stability. Numerous cell lines derived from choriocarcinomas, notably BeWo, JEG-3, and JAR, are commonly employed to investigate placental formation and trophoblast fusion. The BeWo cell line was initially isolated from brain metastases of choriocarcinoma in three women in 1959 [29]. Following 304 consecutive transplants, these cells were preserved in hamsters for eight years until they were co-cultured with human meconium tissue in 1968. This pivotal event led to the establishment of the BeWo cell line, capable of producing hCG [30, 31]. In 1979, it was uncovered that treating BeWo cells with methotrexate caused them to differentiate from a CTB-like phenotype to a multinucleated STB-like phenotype [32]. Various exogenous cAMP analogs, including Forskolin (FSK), cholera toxin, 8-Br-cyclic AMP, and di butyryl-cyclic AMP, were then used to trigger BeWo cell fusion. Consequently, BeWo cells underwent morphological changes, extensively fused into multinucleated giant cells, and expressed placenta-specific proteins such as hCG, placental alkaline phosphatase, and SP1 [33]. Since then, the BeWo cell line has become widely utilized for studying the mechanism underlying trophoblast fusion [16, 34], drug transport [35], STB extracellular vesicles (STBEVs) [36], and metabolism [37]. Furthermore, it has served as a valuable model for investigating the pathogenesis and therapeutic effects of various placenta-derived pregnancy disorders, including PE and recurrent miscarriage [38].

JEG-3 and JAR serve as non-classical cell fusion models for investigating trophoblast fusion. JEG-3, a subclone of BeWo, originally derived from the re-injection of BeWo into the hamster bursa [39]. JAR is also isolated from placental trophoblastic tumors and is known for its secretion of various placental hormones [40]. Despite their responsiveness to the induction of cAMP analogs and the production of multiple placental hormones, both JEG-3 and JAR exhibit limited intercellular membrane fusion, thereby failing to undergo substantial fusion [41–43]. E-cad immunofluorescence staining and the two-color fluorescence cytoplasmic dye assay also showed that the formation of syncytia was limited to BeWo cells rather than JEG-3 and JAR cells [25, 44].

Over recent decades, these cell lines have played a pivotal role in advancing our understanding of STB formation. Notably, several transcription factors, such as BCL6 [45], OVOL1 [21], HDACs [46], p57, p21, CREB [47], DLX3 [48], GATAs [49], TBX3 [50], and various signaling pathways including cAMP/PKA [51], cAMP/Epac1/CaMK1 [52], PI3K/mTOR [53], JAK/STAT [54], Wnt [55], MAPK [56], have greatly benefited from these cell line models. Additionally, studies on factors related to cytomembrane remodeling, such as E-cad [57], calponin-3 [58], syncytin-1, and syncytin-2, has also been greatly advanced by these cell line models. However, caution should be exercised when employing these cells, as they exhibit heterogeneity and have undergone more than 300 passages within hamster capsules, resulting in various chromosomal aberrations. Moreover, their genome-wide DNA methylation pattern markedly differ from those of primary trophoblasts [59]. Therefore, it is advisable to complement the findings from these cell studies with corroborative evidence obtained from PHTs.

## Primary human trophoblast

PHTs isolated from the human placenta are widely recognized as a robust model for investigating trophoblast fusion. In 1986, high-purity functional CTBs were isolated from human term placenta using Percoll gradient centrifugation based on the standard trypsin-DNAase dispersion protocol. These isolated CTBs spontaneously differentiated and formed multinucleated syncytial trophoblast-like cells. Subsequent in vitro culturing for 24–48 h led to a remarkable 90% fusion rate within 72 h [60]. Douglas et al. subsequently conducted a procedure that completely removed HLA-G^+^ EVT cells and vimentin^+^ stromal cells from PHTs using an immunomagnetic separation technique [61]. Alternatively, PHTs can be isolated from early pregnancy placenta through Percoll gradient centrifugation, coupled with EPCAM, ITGA6, or EGFR enrichment [62, 63]. These PHTs maintain the same ability for spontaneous cell fusion in vivo, and the fused PHTs exhibit a notable secretion of placental hormones, such as hCG and hPL [64]. The PHT model has been widely employed for characterizing numerous pivotal regulatory genes involved in the trophoblast fusion process [21], including YAP [65], ID2 [66], HDACs [46], TFAP2 [67], DLX3 [68], GATAs [49], ZO-1 [22], and Cx43 [69]. Beyond these applications, the PHT model has also served as a valuable model in the examination of the anti-infection effects of the placental barrier against microorganisms [70], the release of STBEVs [36, 71], and drug transport [72].

In most cases, the isolated PHTs are prone to contamination by stromal cells and blood cells. Despite PHTs are prone to spontaneously forming STB, they fail to form a completely intact monolayer in a monolayer culture system, making it challenging to use for barrier studies. Furthermore, PHTs exhibit limited or no proliferative capability. Fused PHTs undergo apoptosis after five days of in vitro culture, necessitating the recurrent isolation of fresh tissue for each experiment. To address this issue, many laboratories now isolate a larger quantity of PHTs and perform additional cryopreservation to ensure their availability for future use. Since the mechanism of trophoblast fusion is varies in early and late pregnancy, PHTs derived from distinct pregnancy stages need to be selected based on the specific demands of their experiments. It is crucial to acknowledge that the PHT model remains an in vitro cell model, and it may deviate from actual placental development because the placental structure and the physiological cell microenvironment in vitro differ from those in vivo [73].

## Placental explants

In the 1990s, the utilization of human placental explants emerged as a model for investigating trophectoderm proliferation and differentiation [74, 75]. Initially, this model was primarily employed to reconstruct the cell column and EVT differentiation, with hCG secretion merely serving as an indicator of placental explant activity [76, 77]. However, in 1997, Palmer et al. reported that the STB layer would degenerate gradually during culture and form a new STB layer within 48 h [78]. They further demonstrated that the hCG secretion from placental explants was symptomatic of the presence of syncytial trophoblasts. Currently, two primary methods of placental explants culture are in use. The first involves directly culture of villous tissue, while the second involves the digesting the outer layer of STB, followed by induction to regenerate a new STB layer using FSK, commonly referred to as “villus digestion-reconstruction” [79]. Subsequently, the natural or digested placental villi are transplanted onto type I collagen or extracellular matrix (ECM) substrates, such as Matrigel, to facilitate subsequent culture. Typically, they are cultured in 2–8% oxygen, which is most suitable for villi growth [80]. Studies have demonstrated that placental explants can produce large amounts of hormones, such as hCG and hPL, within a few hours of culture [81].

Placental explants preserve the structure of chorionic tissue and multicellular crosstalk, rendering them a promising avenue for investigating the cellular metabolism of the human placenta. Particularly, explants derived from placentas with clinically known pathologies, such as FGR or PE, hold potential for enhancing our comprehension of the enduring impact of these pathologies on STB renewal and hormone secretion. However, these explants cannot be cultured for extended periods, and chorionic tissue tends to exhibit cell swelling, fragmentation, and disruption of STB cell junctions after 24 h of culturing [82]. Nonetheless, after 48 h, the bottom cytotrophoblast forms a new STB, making it a suitable model for studying the mechanism and morphology of CTB differentiation to STB. When studying the function of STB as a component of a placental barrier, the non-degraded STB cultured within 24 h is preferable as it better characterizes the structure and function of STB in vivo. In 2021, Nadja et al. established a closed flow system for mimicking the utero environment. The flow-cultured tissue in this bioreactor retained better placental tissue viability and structural integrity during 48 h culture, suggesting it as a more suitable method for mimicking in vivo vesicle release from the STB into the maternal circulation [83]. However, placental explants comprise various cell types, including mesenchymal cells, endothelial cells, blood cells, and placental immune cells. Thus, it is challenging to assess or manipulate gene expression in the manner of a single-cell type. Additionally, the proliferation ability of placental trophoblasts decreases significantly with gestational age. Consequently, the selection of placental explants from different gestational ages should be contingent on the specific experimental requisites and objectives.

## Human trophoblast stem cells

Common trophoblast fusion models are limited in use because they cannot self-renew (such as PHT and explants) or have heterogeneity (such as choriocarcinoma cell lines). Human trophoblast stem cells (TSCs) are an emerging in vitro model for the study of trophoblast cells. These cells have the normal karyotype and exhibit the capacity for unlimited proliferation and multi-directional differentiation when cultured in vitro. Currently, the utilization of TSCs for investigating placental trophoblast differentiation has become a research hotspot. The TSCs can be derived from two primary sources: (i) human placental trophoblastic tissue and (ii) human pluripotent stem cells (PSCs) including human embryonic stem cells (ESCs), human induced pluripotent stem cells (iPSCs) and human expanded potential stem cells (ePSCs). This section focuses on describing the feasibility, advantages, and disadvantages associated with these different sources of TSCs as trophoblast fusion models.

## Human trophoblast stem cells derived from placenta

In 2018, Okae et al. successfully isolated proliferative CTBs from first-trimester placental tissue. These isolated cells were subsequently cultured in a specialized TSC medium, leading to their differentiation into human trophoblast stem cells (TS^CTB^ cells), which can maintain self-renewal and be cultured over time [84]. The TS^CTB^ cells expressed trophoblast markers, including GATA3, TP63, TEAD4, and C19MC miRNA. Moreover, the TS^CTB^ cells fused effectively into large syncytia and highly expressed STB markers hCG and SDC1 when induced by FSK. RNA-seq revealed that TS^CTB^ cells had a similar transcriptome to PHT in early pregnancy. Subsequently, the term placental villous cytotrophoblasts (vCTBs) were also reprogrammed into induced trophoblast stem cells (iTSCs) [85]. These iTSCs not only expressed characteristic TSC markers, such as GATA3, TP63, and TFAP2C but also exhibited markers associated with proliferative epithelial cells, including ITGA6, E-cad, and CK7. Remarkably, iTSCs have the capacity to differentiate into multinucleated STB-like cells capable of secreting hCG upon FSK induction [85]. Compared to iPSCs-derived iTSCs, term placental tissues can be directly reprogrammed into iTSCs, reducing the risk of tumorigenicity by avoiding multi-step induction [86, 87]. However, iTSCs obtained by reprogramming have the potential for retroviral integration, which limits their application in clinical or therapeutic applications.

In 2022, CTBs isolated from term placentas were cultured in hypoxia condition (1% O_2_) to successfully establish trophoblast stem cells (TS^term^ cells) [20]. TS^term^ cells exhibit similar characteristics to TS^CTB^ cells that can produce hCG^+^ multinucleated STBs-like cells under the induction of FSK or A83-01. Compared with the first-trimester villus, the term placenta can be obtained directly after delivery without invasive manipulation. Moreover, term placental tissue is accompanied by pregnancy history and outcomes, suggesting that iTSCs derived from it could potentially recapitulate the development of pregnancy-related diseases. Damage caused by reactive oxygen species and DNA methylation accumulated in the placenta throughout pregnancy [88, 89]. In this context, iTSCs serve as a valuable model for investigating placental injuries. Many stem cell populations exhibit the phenomenon of efflux fluorescence dye Hoechst 33342, resulting in a “side population” of cells that can be isolated by flow cytometry [90]. The side-population technique enables the direct isolation of a specific population of human candidate TSCs population (cTSCs) from placental tissue [91, 92]. The cTSCs can be expanded and passaged in TSCs medium, allowing them to differentiate into multinucleated hCG^+^ and syncytin-1^+^ STB without long-term in vitro culture [93]. Therefore, the utilization of side-population technology offers a complimentary approach to enhance our understanding for the TSCs model.

The TSCs maintain normal karyotypes and can be subcultured in vitro many times, exhibiting the capability to fuse into multinucleated STB-like cells. Currently, the TSCs model is extensively employed for investigating the differentiation of CTBs and elucidating specific cell markers and human specific transcription factors, such as TEAD4 [17] and MSX2 [94], involved in placental progenitor CTBs [94–96]. However, laws in many places strictly restrict the use of tissues obtained from the elective termination of pregnancy, so extracting TSCs from full-term placental tissue is an alternative. However, it is worth noting that the transcriptome profile of iTSCs derived from the term placenta closely resembles that of TS^CTB^ cells but is different from that of PHT derived from the term placenta. Therefore, it remains unclear how much physiological information characteristic of the term placenta is preserved in iTSCs from term placenta.

## Human trophoblast stem cells derived from human pluripotent stem cells

The PSCs include primed and naïve ESCs, iPSCs, and ePSCs. These cells can maintain unlimited proliferation and multi-directional differentiation in vitro and can differentiate into almost all cell types. The methodologies for generating and cultivating PSCs have been comprehensively examined in several reviews [97, 98].

In 2002, BMP4 was employed to induce differentiation of primed ESCs into multinucleated STB-like cells capable of secreting hCG, progesterone, and estradiol-17β [99]. With the emergence of naïve ESCs-derived conditions, several studies used the TSCs medium [84] to convert naïve ESCs into TSCs (nTSCs) [100]. Characterization of the nTSCs revealed trophoblastic lineage traits, including positive expression of ITGA6, EGFR, CK7, TEAD4, and TP63, along with the absence of CDX2 expression [101]. Under the induction of FSK, the nTSCs further differentiated into multinucleated STB, accompanied by the expression of SDC1 and hCG secretion [102]. Scanning electron microscopy examinations illustrated that STBs derived from nTSCs displayed similar microvilli as those observed in vivo [100]. RNA-seq further revealed that the global gene expression profiles of ESCs-derived TSCs and STB resembled those of TS^CTB^ cells and differentiated STB, respectively [101, 103]. STB derived from ESCs expresses a variety of transporters, rendering them a valuable in vitro model for investigating placental barrier function, placental transport, and metabolic activities [104]. Human iPSCs, reprogrammed from somatic cells, can be induced into iTSCs that closely resemble TS^CTB^ cells in both molecular and functional aspects [86, 105]. These iTSCs have the capacity to form multinucleated syncytia and secrete estradiol and hCG upon induction with cAMP/FSK [106]. Notably, iPSCs derived from umbilical cord-derived mesenchymal stem cells, obtained from placentas of pregnancies with or without PE, have been successfully established. Trophoblast cells derived from PE-iPSCs demonstrated deficiencies in syncytialization and a diminished response to hypoxia, offering a valuable model for studying trophoblastic fusion disorders associated with PE [107]. Somatic cell reprogramming presents advantages over ESCs in terms of cell acquisition and histocompatibility. Moreover, when ePSCs are cultured under TSCs culture conditions, they can be transformed into TSCs. TSCs derived from ePSCs express various trophoblast transcriptional regulators, such as GATA3 and TFAP2C, and can differentiate into SDC1^+^ multinucleated STB [108].

Transcriptomic analysis has revealed that TSCs derived from PSCs closely resemble CTBs during the early stages of embryo implantation [101, 103], making them ideal for studying early trophoblast lineage development [109]. Furthermore, PSCs can undergo effective genetic modification, offering a valuable avenue for exploring the roles of specific factors in trophoblast fusion. Imprints play a pivotal role in placental development, and certain parental imprints remain only in the placenta. However, culturing cells under ESCs and iPSCs conditions can lead to widespread imprint erasure, which is very different from the normal human placenta [103, 110].

## Three-dimensional cell spheres

Although monolayer cell lines remain an essential tool for studying trophoblast fusion, their monolayer and flat growth patterns substantially deviate from the 3D villi structure in vivo. This divergence significantly constrains their utility in research pertaining to villus histogenesis, cell polarity, and tissue remodeling. To address this issue, various 3D cell culture models have been developed.

In 2005, the SGHPL-4 cells were incubated with Cytodex-3 microcarrier beads, followed by transfer into the rotating wall vessel (RWV) bioreactor to study trophoblast differentiation and invasion. The RWV bioreactor creates a low-shear culture environment, promoting cell-to-cell adhesion and the formation of a 3D structure that closely resembled a more “tissue-like” phenotype [111]. In 2016, JEG-3 cells, attached to Cytodex-3 beads, were co-cultured with human brain microvascular endothelial cells (HBMECs) in an RWV bioreactor to produce 3D cell spheres [112]. Notably, these 3D JEG-3 cell spheres exhibited significantly elevated expression levels of STB markers, including hCG, hPL, PP13, and MFSD2, surpassing even those observed in PHT cells. Interestingly, 3D JEG-3 cell spheres displayed a high rate of spontaneous fusion and featured dense brush-like borders. RNA-seq analysis also indicated that the transcriptional profile of 3D JEG-3 cells closely resembled that of primary syncytial trophoblast cells. Furthermore, 3D JEG-3 cells possessed a similar immune barrier to the STB layer and were highly resistant to vesicular stomatitis virus (VSV) infection and toxoplasma gondii infection [112]. Fully differentiated 3D JEG-3 cells were also reported to be resistant to trichomonas carinii infection. In 2018, following the derivation of TSCs, 3D trophoblast stems cell spheres (3D-TS) based on low adhesion plates were successfully established. 3D-TS spontaneously formed a cyst-like structure secreting hCG, which further enhanced the formation of cyst structure under the induction of FSK [84]. Subsequently, nTSCs were able to established hCG^+^, SDC1^+^, and TEAD4^-^ STB typical cyst-like cell spheres using this system [101]. Li et al. also achieved 3D JEG-3 spheroids based on an ultra-low attachment culture system, and FSK could induce substantial spheroids fusion [113]. Another 3D culture model is embryoid bodies (EBs), capable of differentiating into the three embryonic germ layers. In 2004, Gerami-Naini et al. employed EBs as an in-vitro model for early embryonic development, successfully differentiating ESCs into trophoblast-like cells [114]. The suspension-cultured EBs could secrete hCG, estradiol, and progesterone. Subsequently, several research groups successfully derived EBs under the conditions of a low adhesion plate or semi-solid medium, and the cells around EBs secreted hCG and expressed STB markers [115, 116]. Recently, TSCs were cultured into a placental sphere model with a high degree of consistency in size and shape using an embryoid body culture plate, and the hCG^+^ STB layer was located at the periphery of the sphere [117].

The 3D trophoblast fusion models provide a better simulation of 3D structure and multicellular complexity of tissue, meanwhile serving as a platform for studying drug and nutrient transport, pathogen infection, and STBEVs release [118, 119]. In the 3D sphere model, the newly formed STB is located outside the sphere, which resembles in vivo morphology. However, some areas may require further attention in the future, including (i) the establishment of a TSCs sphere model of multicellular co-culture; (ii) addressing the issue of cells in the center of the sphere, which are prone to apoptosis due to the lack of nutrition.

## Organoids

The spheroidal system is a fundamental 3D culture system that offers a more realistic and physiologically relevant model compared to the monolayer system in vitro. However, it is not feasible to maintain it for long-term culture, and it still cannot completely simulate the complexity of the placenta. In recent years, the emergence of a 3D placental organoid culture system has provided a promising platform for studying trophoblast fusion. In 2018, two independent groups successfully established CTB organoids (CTB-ORGs) using vCTBs obtained from first-trimester placental tissue [120, 121]. Remarkably, CTB-ORGs exhibited long-term viability, with cultures extending for over five months without any observed abnormal epithelial-mesenchymal transitions during repeated passages. The CTB-ORGs not only proliferated and self-renewed in vitro but also underwent spontaneous cell fusion to the center to produce functional multinucleated STBs and secrete hCG. Additionally, electron microscopy unveiled microvilli formation on the multinuclear structure. RNA-seq, DNA microarray, and genome-wide DNA methylation analysis further revealed similar features between CTB-ORGs and first-trimester placental villi. In 2021, the protocol for CTB-ORGs development was used to generate 3D JEG-3-ORGs, capable of differentiating into STB and EVT [122]. In 2022, nTSCs were used to establish the 3D stem-cell-derived trophoblast organoids (SC-TOs), containing multiple trophoblast properties closely mirroring those of CTB, STB, and EVT cells found in human post-implantation embryos. SC-TOs also demonstrated selective susceptibility to SARS-CoV-2 and Zika virus infection, rendering them a promising model system for placental infections [123]. Recently, a study successfully constructed trophoblastic organoids (TOs) using Ki67^+^ trophoblast cells isolated from the second and third trimesters of human pregnancy (26-41 weeks gestation) [124]. The TOs were inward-facing fused to STB, expressing SDC1 and secreting hCG. Many congenital infections occur in the second trimesters and third trimesters of pregnancy, and TOs isolated from later stages of pregnancy can serve as a better model for investigating the mechanisms of microbial vertical transmission and antiviral innate immune signaling.

The 3D trophoblast-like ORGs are not limited by cell numbers, can proliferate continuously, and can be passaged, cryopreserved, and thawed for continued culture [125, 126]. Trophoblast-like ORGs can differentiate into STB and EVT, mimicking the developmental process of placental villi in vivo [65]. However, it is noteworthy that in trophoblast-like ORGs, the proliferative vCTBs are formed at the outer Matrigel region, while the formation of STB occurs within the luminal space, which differs from the direct contact between STBs and maternal blood on the surface of villi in vivo. TOs derived from either first-trimester or later stages of pregnancy trophoblast primarily represent the expression profile of first-trimester placental tissue, and they may not be suitable as a cell fusion model to study the third-trimester placenta. The placental ORGs model remains an ongoing exploration, and the use of various cytokines and pathway inhibitors/activators within the culture system is still at an experimental stage.

## Placenta-on-a-chip

The unique physical properties of microfluidic devices allow for precise control over cell arrangement and subcellular environments, providing the foundation and system for the organ-on-chip model [127]. In 2015, Miura et al. developed a microfluidic device that mimicked the placental barrier, consisting of two polydimethylsiloxanes (PDMS) microchannels separated by a vitrified collagen (VC) membrane to simulate maternal (upper) and fetal (bottom) blood circulation [128]. In 2016, Lee et al. created a dual-chamber co-culture model using JEG-3 cells and umbilical vein endothelial cells (HUVECs), seeding them into two PDMS microchannels separated by a thin ECM membrane [129]. The “placenta-on-a-chip” microdevice provides a new opportunity to model and analyze the placental barrier. Since then, several studies have used BeWo or JEG-3 cells to co-culture with HUVECs to establish a placental microchip system, which enables the emulation of material exchange process in vivo and serves as valuable tools for studying substance transport [130], metabolisms [129, 131], and anti-infection of the placental barrier [132, 133]. In 2019, Nishiguchi et al. also preliminarily achieved a multilayer culture of primary CTB, primary fibroblasts (normal human dermal fibroblasts, NHDF), and HUVECs by using the capillary model composed of nano-films [134]. In 2022, a 3D model of placental trophoblast from hiPSCs was successfully established using a perfused microfluidic chip [135].

The placenta-on-a-chip enables the reconstruction of the multilayer structure of the placental barrier and the hemodynamic environment [136]. Multicellular co-culture and fluid shear stress environment within this system more closely simulate the environment in vivo than the static culture. Fluid shear stress triggers the formation of microvilli in the human placental trophoblast [128]. Compared to the static culture, the microarray placenta exhibits more complete and dense microvilli protrusions at the top of trophoblast cells. Additionally, this multicellular co-culture allows for the study of intercellular communication and its impact on placental function, significantly advancing our understanding of the structure-function relationships within the organs. Miniaturization of the system will greatly reduce the number of various reagents and allow for real-time monitoring of dynamic changes in all types of data, resulting in cost savings for research [137, 138]. The placenta-on-a-chip has been used to study trophoblast invasion, toxicological screening, microvilli formation, placental pathology, and placental exosome capture. However, to achieve mature technology and low-cost large-scale application, improvements are needed in areas such as liquid flow control, monitoring technology, and co-culture systems involving a wider range of cells and microorganisms.

## Conclusion

### The issues faced by the trophoblast fusion models

Although models used to investigate the mechanisms of human trophoblast fusion are constantly improving, the existing models still encounter several issues. (i) The STB undergoes dynamic changes throughout pregnancy, and various CTB fusion models exist. However, a significant limitation is the absence of clear molecular markers to define the scope of applicability of the existing models. (ii) The microenvironment of the maternal-fetal interface plays a critical role in regulating trophoblast fusion. Unfortunately, most existing models do not adequately consider this critical factor. For instance, the uterine environment during pregnancy typically maintains an oxygen tension of 2–8%, yet the majority of experiments are conducted under culture conditions exposing cells to atmospheric oxygen levels (approximately 20% O_2_). (iii) While significant progress has been made in the study of PSCs and organoids for trophoblast differentiation in recent years, the culture systems, cell sources, and control over differentiation direction are still in the exploratory stage, with the added challenge of high culture costs. (iv) Most available cell fusion models rely on biochemical reagents, such as FSK, for cell fusion induction, which may differ from the spontaneous fusion of placental trophoblast in vivo or PHT in vitro. (v) TSCs can be maintained as CTB-like cells within a specific culture system by employing a range of cytokines and pathway inhibitors. Nevertheless, the presence of cells in a comparable state in an in vivo setting remains uncertain. Genome-wide analyses have also revealed that TSCs do not exhibit transcriptional similarity to trophoblasts in vivo [139]. Notably, recent studies have demonstrated that these ePSCs lack extensive expanded or extended potential and do not possess the ability to differentiate into the trophoblastic lineage [98, 140, 141]. Furthermore, the TSCs, regardless of their origin from ESC, iPSC, or placenta tissue, have not undergone rigorous evaluation regarding their similarity and differentiation potential compared to true trophoblast cells. Thus, there is an ongoing need for thorough characterization and optimization of methods derived from TSCs.

### Future perspectives

Through the systematic evaluation of existing models, there is still a gap between them and the actual differentiation process in vivo. Single-nucleus RNA-sequencing (snRNA-seq) has been used to determine the degree of transcriptional diversity within multinucleated skeletal myofibers [142]. This technique has been successfully applied to both mouse placentas and trophoblast lineages derived from ESCs [143, 144]. The application of snRNA-seq in elucidating unique transcription profiles of the STB presents a novel avenue for investigating trophoblast fusion. The crosstalk between CTBs and the maternal-fetal microenvironment is an important factor in determining their lineage differentiation. Consequently, unraveling the regulatory mechanisms governing signal transduction from the maternal-fetal microenvironment to the intracellular signaling network offers promise in constructing a spontaneous fusion model that closely resembles in vivo conditions. Placenta-on-a-chip is a potential platform for simulating the structure and function of placental villi in vivo, enabling multicellular co-culture and real-time monitoring, as well as precise microenvironment control. However, substantial efforts are still necessary to reduce both the threshold and cost associated with this technology.

## Data Availability

Data sharing is not applicable to this article, and all data are included in the manuscript.
